# Instability of the perceived world while watching 3D stereoscopic imagery: A likely source of motion sickness symptoms

**DOI:** 10.1068/i0647

**Published:** 2014-10-07

**Authors:** Alex D. Hwang, Eli Peli

**Affiliations:** Department of Ophthalmology, Schepens Eye Research Institute, Massachusetts Eye and Ear, Harvard Medical School, Boston, MA, USA; e-mail: alex_hwang@meei.harvard.edu; Department of Ophthalmology, Schepens Eye Research Institute, Massachusetts Eye and Ear, Harvard Medical School, Boston, MA, USA; e-mail: eli_peli@meei.harvard.edu

**Keywords:** 3D perception, motion sickness, stereoscopic display, simulation sickness, 3D display, 3D television

## Abstract

Watching 3D content using a stereoscopic display may cause various discomforting symptoms, including eye strain, blurred vision, double vision, and motion sickness. Numerous studies have reported motion-sickness-like symptoms during stereoscopic viewing, but no causal linkage between specific aspects of the presentation and the induced discomfort has been explicitly proposed. Here, we describe several causes, in which stereoscopic capture, display, and viewing differ from natural viewing resulting in static and, importantly, dynamic distortions that conflict with the expected stability and rigidity of the real world. This analysis provides a basis for suggested changes to display systems that may alleviate the symptoms, and suggestions for future studies to determine the relative contribution of the various effects to the unpleasant symptoms.

## 1 Introduction

People view the three-dimensional (3D) physical world without any particular discomfort. However, viewing stereoscopic displays frequently causes complaints of visual discomfort, including symptoms closely resembling motion sickness.

Visual discomfort symptoms such as eye strain, blurred vision, and double vision may be related to fundamental limitations of stereoscopic 3D (S3D) display technology that result in oculomotor conflict between accommodation and convergence demands (Hoffman, Girshick, Akeley, & Banks, 2008; Howarth, 2011; Lambooij, IJsselsteijn, & Heynderickx, 2007; Peli, 1995; Rushton & Riddell, 1999; Shibata, Kim, Hoffman, & Banks, 2011; Tam, Speranza, Yano, Shimono, & Ono, 2011; Ukai & Howarth, 2008; Wann, Rushton, & Mon-Williams, 1995); a difference between viewing distance to the screen, *focal distance*, and the distance to the virtual stereo images, *convergence distance* (Yano, Emoto, & Mitsuhashi, 2004). Lack of naturally occurring blur at different virtual distances may also result in visual discomfort (Wöpking, 1995). Studies of 3D visual discomfort with stereo displays (Pocket, Salmimaa, Polonen, & Hakkinen, 2010; Woods, Docherty, & Koch, 1993) have suggested that 2D image distortions during capture (e.g., lens distortion) and projection (e.g., keystone distortion) cause localized misalignment between the left and right eye images, especially in the periphery. These distortions disrupt natural stereo fusion, and may increase overall 3D viewing discomfort (see Lambooij, Fortuin, Heynderickx, & IJsselsteijn, 2009 for review). A variety of potential causal factors for visual discomfort are also discussed in Kooi and Toet (2004) and Howarth (2011).

Although a variety of possible factors have been identified as causes of S3D discomfort, motion-sickness-related symptoms experienced during S3D viewing, ranging from light-headedness, dizziness, queasiness, nausea, and vomiting, cannot be explained directly by current understanding of the oculomotor conflict, or lack of relative blur. The S3D-related distortions have been mentioned as possible causes for viewing discomfort, but the discussions were usually limited to static (2D) distortions and their impact on binocular fusion, and have not been explicitly extended to explain how the dynamic changes of distortions may affect viewer's depth perception and cause motion sickness-like discomfort. We postulate that such discomfort must be related to a viewer's perception of S3D motion.

In this paper, we show that (1) the chain of 3D stereoscopic image capture and display causes dynamic depth distortion in stereo image motion, which results in spatiotemporal cue conflicts in depth perception of the displayed environment, and (2) the viewer's head movements cause perceptual instability of the displayed visual world and high-level cue conflicts. We suggest that either or both may account for motion sickness when viewing S3D. We then propose how these might be resolved in specific systems, enabling future rejection or verification of our hypothesis and measurement of the impact of these effects on motion sickness symptoms

## 2 Visually induced motion sickness

Symptoms of visually induced motion sickness (VIMS) are reported in both 2D and S3D presentations (in movies or video games), but it has been reported that S3D stimuli cause significantly higher levels of discomfort than 2D stimuli (Kuze & Ukai, 2008; Solimini, 2013; Ujike & Watanabe, 2011). A linkage of S3D and VIMS has been suggested in Howarth (2011), but no direct explanation was provided for why S3D would cause VIMS.

VIMS is generally explained as a physiological response elicited by *sensory conflicts* between two different motion signals (Guedry, 1970; Irwin, 1881; Money, 1970), usually one from the vestibular system and the other from the visual system (Kennedy, Drexler, & Kennedy, 2010), although other sensory combinations are possible (e.g., auditory). This conflict of motion signals is presumed to cause perceptual stress during the information integration process, and provoke poison response-like symptoms such as light headedness, spatial disorientation, nausea, and purging of stomach contents. Similar conflict may occur when a person watches a 2D movie that induces a strong self-motion signal (an abrupt camera motion, for example), while the vestibular system generates almost no self-motion signal (since the viewer is at rest). These two sources of motion information, which are well-synchronized during normal activity in natural environments, need to be reconciled by the brain to make a correct judgment of self-motion and positioning to maintain postural stability. When one signal contains strong self-motion and the other signal produces little self-motion, the information conflict invokes motion sickness symptoms. The accumulation of such contradictory signals over time may trigger severe VIMS.

As an extension of the original sensory conflict theory, the *sensory rearrangement* theory was proposed (Reason, 1970; Reason, 1978; Reason & Brand, 1975). This theory includes a wider range of possible causes of conflicts by introducing the concept of a sensory comparator that matches the current sensory inputs with a stored trace of motion information expected based on previous experiences with the spatial environment; the *exposure-history* (Held, 1961). In this theory, if there is a discrepancy between the present sensory input and the exposure history, a mismatch signal is generated and may trigger motion sickness symptoms. Continuous interactions with such nausiogenic stimuli eventually register the new experience as a new type of sensory pattern, which needs to follow its own sensory integration rule. This sensory rearrangement and comparator adaptation results in the eventual dissipation of VIMS symptoms. Note that the original sensory conflict theory did not include an adaptation mechanism, and could not explain why motion sickness symptoms can be overcome by repeated experience (e.g., by sailors and pilots).

Causes of motion sickness are not limited to the low-level *inter-sensory* conflicts between visual and vestibular signals. A study of VIMS in helicopter flight simulations (Miller & Goodson, 1958) suggested that the problem may be caused by some combination of dynamic distortions in the projected scenery, visual to visual *intra-sensory* conflict, where the perceived directional changes and angular rate of motion contain dynamic errors.

The sensory rearrangement theory can be applied to explain everyday experiences. For example, when new spectacles are fitted, people sometimes experience nausea, vertigo, and other motion-sickness-related symptoms for the first several days of wear, presumably due to perceived distortion created by the prismatic (or other optical) effects of refractive lenses (Moodley et al., 2011; Tuan & Jones, 1997). Dynamic distortions of the retinal image as subjects move (whether or not the lens corrects their vision) expose subjects to unaccustomed optic flows that violate the expected natural optic flows that have been built up throughout previous experiences. These effects are even more common and more dramatic with first fitting of progressive addition lenses that by design induce larger magnitude peripheral distortions than single-vision spectacle lenses. These symptoms subside as the wearers adapt to the new form of optical flow. This requires sensory rearrangement or adaptation, so that orientation and speed changes of unnatural optical flow are remapped to be perceived as natural. Returning to the old spectacle correction may cause a brief return of the discomfort, but with repeated alternation, people are able to switch between these optical corrections (e.g., between contact lenses and spectacles) with no difficulty.

Perception of visual unnaturalness can arise from various motion cue conflicts. Under natural viewing conditions, the perceived size of an object follows linear perspective rules (e.g., an object moving toward the viewer enlarges in retinal projection), and the visual system utilizes this to estimate object motion in depth. However, in movies, this natural expectation of size change can be manipulated by camerawork, as in *contra-* or *Hitchcock-zooms* (Barbera, 2005). If a scene is recorded with a camera moving toward a near object, while applying a zoom-out to keep the near object's angular size constant, this camerawork creates continuous perspective distortion, wherein the background scene appears to stretch backward while the size of the near object is kept constant. This creates perceptual inconsistency with purportedly rigid depth structure appearing to expand in depth, and the viewers of the scene experience strong visual cue conflicts. This technique was used in the movie *Vertigo* (1958) to simulate the main character's perceptual disorientation when looking down the tower stair shaft.

A study of S3D movie spectators found that about 1/4 felt motion sickness symptoms and the symptoms lasted more than 2 hours after viewing the movie ended (Solimini, Mannocci, & Di Thiene, 2011). Yang et al. (2011) reported that the motion-sickness-related symptoms, such as dizziness, nausea, and disorientation, are greater with S3D than 2D, young viewers compared to old viewers, females compared to males, and from a central rather than an oblique seat location. Studies of non-stereoscopic 2D virtual reality (VR) environments conjectured that the apparent fidelity of more-immersive environments increases perceptual reality, and consequently intensifies motion sickness (Cobb, 1999; Kennedy & Lilienthal, 1995). Although the increased perceptual reality of visual stimuli may explain to some degree the overall promotion of S3D motion sickness symptoms, it does not explain the S3D-specific motion sickness issues, as nothing is more real than the real world itself. These must be related to distortions of motion in depth. To our knowledge, there has been no specific mechanism proposed for the causes of motion sickness due to S3D motion. In this paper, we are suggesting that content-based scene motion in a distorted depth space, and the perceptual instability induced by the viewer motion, causes high-level visual–visual cue conflicts and that either may be a cause of S3D motion sickness.

## 3 Depth perception

Monocular depth cues, such as occlusion, shading, motion parallax, accommodation, familiarity with object size, linear perspective, and texture density change, are effectively employed in depth perception. However, we perceive the world with two eyes, and our depth perception strongly relies on horizontal angular disparity of images falling on the retinas; *stereo depth cues*. In the physical world, monocular and stereo depth cues are in perfect agreement, and they are combined to provide perceptual estimates of depth differences (Cutting & Vishton, 1995). Current S3D technology mainly uses depth-by-disparity (stereo) to add stereo depth cues. Two cameras are used to capture slightly different views of a scene, those captured scenes are separately presented to the corresponding eyes, and the viewer's brain extracts stereo depth information from the presented angular disparity and integrates it with monocular depth information available in the 2D images to perceive a consistent 3D world.

However, as we show below, the stereo and monocular cues provided in this way are not always consistent, given the common S3D image capture/display/view process. These inconsistencies are a source of vision-to-vision conflicts that may be the cause of the VIMS associated with viewing S3D. These inconsistencies are more apparent with scene or viewer's motion.

### 3.1 Depth-by-disparities

**Figure 1.**
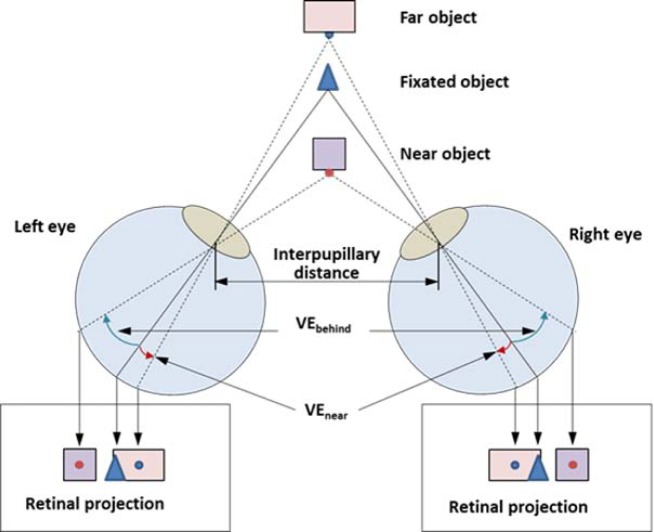
Horizontal visual eccentricities (VE) of objects projected to each eye. Note that the positive or negative VE is measured as clockwise or counter clockwise rotation from the center of visual field (fovea), respectively.

When a person fixates on an object in space, both eyes are directed to that point, and the projection of the fixated point falls onto the fovea in each eye. This alignment is required to avoid diplopic (double) vision. If the fovea is assumed to be the center of the visual field, all other points in the space can be referenced via angular polar coordinates, with the radial distance known as the *visual eccentricity* (VE). For simplicity, the discussion herein focuses on points lying on the horizontal plane through the nodal points of the eyes, with no vertical offsets. In addition, since the two eyes are separated horizontally by the *interpupillary distance* (IPD), the objects located in front or behind the fixated point will be projected at different visual eccentricities in each eye (see Figure 1). In this case, the *angular disparity* (AD) of a point can be defined as the difference between the visual eccentricities of the points projected on the left and right eyes. In other words, if we follow the fovea-centric angular coordinates, an AD can be computed by subtracting the VE of an object on the left eye from the VE of the same object on the right eye. As a result, the objects near/far relative to the fixated distance (along the direction of fixation) get positive/negative AD, respectively. Note that since the fixated point in space is projected onto the reference position (fovea) of each eye, which are both at zero VE, the AD for the fixation point is also zero. In other words, the AD encodes the relative distance from the viewer to objects in the direction of the fixated object, referenced to zero at the fixated distance.

This also means that if the perception of object depth is assumed to be solely determined by the AD on viewer's retinas, we can estimate viewer's perceived relative depth changes by computing the AD changes of the objects throughout the series of spatial mappings of the 3D display processing chain: scene capture (3D to 2D), image processing (2D to 2D), projection (2D to S3D), and perception (S3D to 3D).

### 3.2 Sample scene configuration and modeling of viewer's depth perception

**Figure 2.**
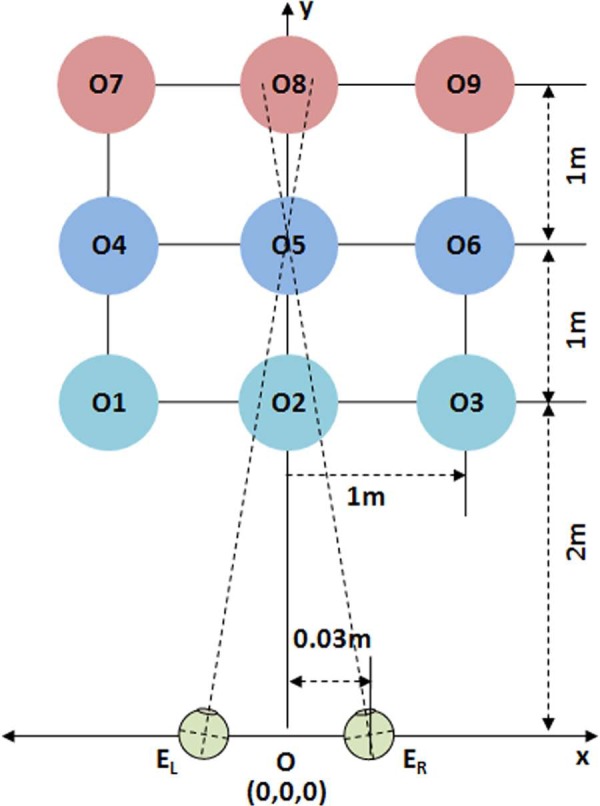
Sample scene configuration in world coordinates. The objects of interest are point objects at the intersections of the grid lines. Note that this and the following diagrams are not to scale.

To illustrate the distortions of depth perception that may be caused by common S3D display processing, we will use a simple array of objects in real space, where the positions of the objects in the real 3D space are defined as in Figure 2. Objects 1–9 are arranged to be on an equally spaced rectilinear grid (in depth).

The origin of the world coordinates is located at the midpoint between the nodal points of the two eyes, and the distance to the nearest row of the grid from the origin is 2 m. Objects are spaced 1 m apart both horizontally (*x* axis) and in-depth (*y* axis). The elevations of the objects and cameras are set at the same height (*z* = 0). The eyes are assumed to be separated 0.06 m, making the coordinates of the left and right eyes *E*_L_ = (−0.03, 0, 0) and *E*_R_ = (0.03, 0, 0), respectively. With this initial setup, when the viewer looks at the front object in the center column, the rectilinear grid spans ±27° of the viewer's horizontal visual field. If the viewer looks at the left or right object in the front row, the grid spans from 0 to 54° or 0 to −54°, respectively. Since there is no elevation difference among objects and eyes, the 3D depth computation is a simple problem in planar geometry, where the VE of an object depends only on a value along the horizontal axis.

### 3.3 Model of AD-based depth perception in human vision system

With the given sample scene configuration, the VE of an object *O*_i_ projected to the left and right eye retinas, while fixating on an object (*O*_F_) can be computed simply by subtracting the angle to the fixated object from the angle to the object. The following equations compute VEs and resulting ADs for objects in the scene. The fundamental geometry of binocular stereopsis and other approaches to derive retinal disparity can be found in Howard and Rogers (1995) and Lipton (1993). We provide these formulations here only to set up the parameters and the notations for our own derivations.

(1) $${\mathrm{VE}}_{L,i}=\text{atan}(\frac{O_{i,x}-E_{L,x}}{O_{i,y}-E_{L,y}})-\text{atan}(\frac{O_{F,x}-E_{L,x}}{O_{F,y}-E_{L,y}})$$

(2) $${\mathrm{VE}}_{R,i}=\text{atan}(\frac{O_{i,x}-E_{R,x}}{O_{i,y}-E_{R,y}})-\text{atan}(\frac{O_{F,x}-E_{R,x}}{O_{F,y}-E_{R,y}})$$

(3) $${\mathrm{AD}}_{i}={\mathrm{VE}}_{L,i}-{\mathrm{VE}}_{R,i}$$

where

VE_*L,i*_, VE_*R,i*_: visual eccentricity of object *i*, as seen in the left and right eye, respectively,
*O*_*F,x*_, *O*_*F,y*_, *O*_*i,x*_, *O*_*i,y*_: *x* and *y* coordinates of the fixated object *F* and object *i*, and
*E*_*L,x*_, *E*_*L,y*_, *E*_*R,x,*_ E_*R,y*_: *x* and *y* coordinates of left and right eye nodal points.

For simplicity and clarity, it is assumed that the distance between the nodal points remained fixed with eye rotations and equal to our declared IPD, as it is the nodal point locations that are the origin of visual eccentricity measurements. The magnitude of nodal point offsets with eye rotations and the distances to the objects of interest affects VE by less than 0.1°, and it affects AD by an order of magnitude less, which is far smaller than the AD effects, which are on the order of 1° that will be discussed in more detail below

**Figure 3.**
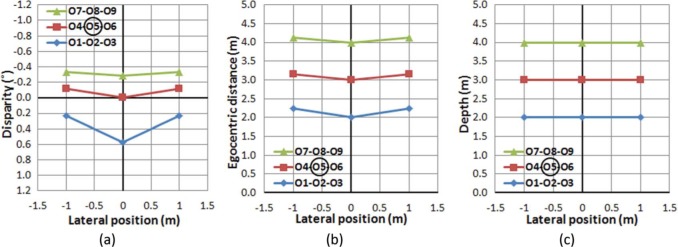
(a) Angular disparity distribution in degrees of objects in the scene seen by a viewer fixating the central object (O5). (b) Physical distance of objects in the scene measured in meters from the midpoint of the line connecting the nodal points of the eyes. (c) Ideal perception of the sample scene.

Figure 3a shows the ADs of the nine objects in the real scene, as projected to a viewer's retinas while fixating at the center object (O5). Note that (1) the fixated object (O5) has zero AD, (2) nearer objects (O1-O2-O3) relative to the fixated objects have positive ADs, and (3) farther objects (O7-O8-O9) have negative ADs. Therefore, ADs in binocular vision can be considered as an encoding of depth structure in “relative” (not “absolute”) distance/depth with respect to the egocentric distance to the fixated object. Along a given line of sight, disparity monotonically decreases with distance, but the scale of the change and the location of zero disparity vary as a function of VE. This suggests that in order to estimate the absolute or actual egocentric distance to an object in the visual field, the human binocular vision system must combine at least two different depth cues, one based on the binocular stereo cues (e.g., AD) that supplies relative spatial layout of the visual scene, and the other based on nondisparity-based visual depth/distance cues (e.g., known size of the aimed object, convergence angle, accommodation, or linear perspective) that provide an estimate of absolute distance to the fixated object. The effectiveness of the various depth cues depends primarily on distance from the viewer to the objects of interest (Nagata, 1993), and combinations among various depth cues are situation dependent (Cutting & Vishton, 1995).

Comparing the result of the AD distribution (Figure 3a) to the egocentric distance distribution of the objects (Figure 3b), it is clear that the objects along the line of sight (0° VE, O2-O5-O8) have larger AD differences than the off-center objects (O1-O4-O7 and O3-O6-O9). Moreover, among the objects on the same line in depth, the nearer objects (O2-O5) show larger AD differences than the distant objects (O5-O8) for the same depth difference (1 m). This means that in order for a viewer to perceive egocentric distance correctly, the AD information acquired by binocular vision has to undergo a nonlinear conversion process (e.g., conversion of Figure 3a–b). Equation (4) applies such conversion to the *egocentric distance* in the physical world from the disparity and eccentricity information of an object in the viewer's visual scene.

(4) $$D_{i}=d*[\cos(\theta_{i})\cot(\omega_{i})+\sqrt{1+\cos^{2}(\theta_{i})\cot^{2}(\omega_{i})]}$$

where

*D*_*i*_: egocentric distance from the viewer to a non-gazed object, *i*,
*d*: distance between nodal points of left and right eyes, inter pupillary distance (IPD),
*θ*_*i*_: VE of the object *i*, relative to the gaze (fixation) direction, taken from the origin, O, and
*ω*_*i*_: AD of the object, *i*.

Once the perceived egocentric distance is computed, it needs to undergo another nonlinear conversion that finalizes the *natural perception of depth*, which is a perceptual judgment that objects on the same frontoparallel depth plane in the physical world are perceived to be at the same depth (e.g., conversion of Figure 3b–c). In the current setup, a viewer should perceive objects on the same row (O1-O2-O3, O4-O5-O6, and O7-O8-O9) to be aligned on the same frontoparallel plane. Equation (5) maps the egocentric distance and VE to the object position in the physical world. Note that the mapping of an off-fovea object only depends on perceived distance and the corresponding VE.

(5) $$P_{i}=D_{i}\cos(\theta_{i})+(\frac{D_{\mathrm{fixated}}-D_{i}\cos(\theta_{i})}{\cos(\theta_{i})})$$

where

*P*_*i*_: perceived depth to the object (distance from the origin to the frontoparallel plane containing the object).

Equations (1)–(5) represent the series of geometric conversions required to perceive the correct depth configuration of the sample scene from the left and right eye VEs of non-fixated objects.

### 3.4 Stability of 3D perception in human vision

Perception of 3D shape (Todd, 2004) and slant (Backus, Banks, Van Ee, & Crowell, 1999; van Ee, Adams, & Mamassian, 2003) are largely unaffected by various (static) perspective conditions and maintain perceived stability of the scene structure. We postulate that stability is necessary to avoid VIMS, and thus it is important to understand how the depth perception of real-world scenes derived from AD structure remains stable throughout (dynamic) eye and head movements.

**Figure 4.**
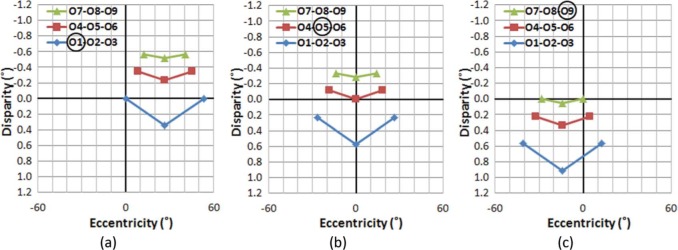
Distribution of ADs and VEs when fixating on different objects: fixating (a) on O1, (b) on O5, and (c) on O9, in the sample scene. Although the absolute AD of objects changes for different fixation conditions, the (relative) AD differences among objects are the same regardless of fixation changes. (Absolute ADs are dependent on the fixated object's location.) Fixated objects are circled in the legends. The vertical axis represents AD in degrees, with negative values representing uncrossed disparities. Depth increases as disparity decreases monotonically. Note that positive/negative disparity and crossed/uncrossed disparity are often mixed in use. While positive disparity means a closer object here (and in graphics/image processing literature), crossed disparity means a closer objects in optometric/clinical literature. Zero disparity, however, does not generally imply that an object is at the same egocentric distance as fixation, unless the object and fixation are the same angle from the midline.

To examine the stability of the AD structure across eye movements, VEs and ADs of objects in the sample scene were computed while assuming that the viewer's head position remains in the center of the world (the origin of our scene) as the eyes fixate different objects in the grid (Figure 4). Note that linear perspective projection was applied to the spatial configuration, so the plots show the VE and AD of each object as seen by the viewer. Thus, we have changed the horizontal axes from the meters of Figure 3a to degrees of visual angle in Figure 4.

If a viewer makes a series of the eye movements from O1 to O5 then O9 (Figure 4a→4b→4c, respectively), it is evident that although the AD of each object changes based on which object is fixated, the AD structure (the shape of disparity plots), which defines relative disparities among objects, is preserved across the eye movements. Therefore, it can be concluded that the viewer's eye movements do not disrupt the perception of the stable (rigid) visual world in natural stereo viewing.

Since the extraocular muscles' proprioception of vergence angle (along with accommodation) provides a relatively inaccurate estimate of absolute egocentric distance to the fixated object, estimating absolute distance to an object needs to rely on non-physiological, but somewhat less reliable, information, such as the known size of the object and size ratios of objects. Therefore, estimating absolute distance must be relied upon only when it is clearly needed. This supports the primacy of relative disparity over absolute disparity for visual perception of the 3D world, as postulated by Westheimer (1979) and implemented by Peli, Hedges, Tang, and Landmann (2001) to eliminate the convergence/accommodation conflict of stereoscopic displays.

**Figure 5.**
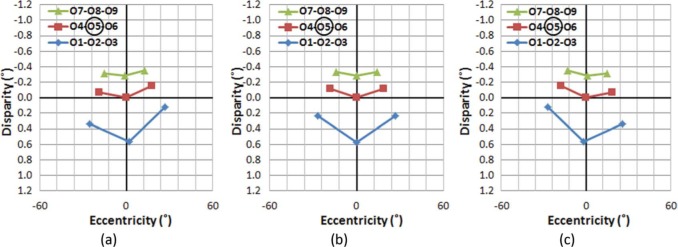
Distribution of ADs and VEs of objects, when head position is at (a) −0.2 m, (b) 0.0 m, and (c) 0.2 m from the origin, while fixating at the center objects (O5) in the physical sample scene.

Similarly, it is necessary to determine if analogous perceptual stability is maintained across viewer's head position changes during natural stereo viewing. This is particularly important because head motion is accompanied by corresponding vestibular motion signals and motion parallax. Figure 5 shows the computed AD structures of the sample scene when a viewer's head is positioned at −0.2 m (left), 0 m (center), and +0.2 m (right) from the center of the world, while the viewer is fixated on the center object, O5.

It can be seen that the AD structure of the scene rotates as a consequence of the shifted head position to reflect perspective rotations. For example, if a viewer is fixating at the center of the grid object (O5), and makes a horizontal head shift from left to right (Figure 5a→5b→5c), ADs of those objects on the left side of the visual field are decreased and the ADs of objects on the right side are increased. Therefore, the viewer will perceive the spatial structure in the sample world rotating clockwise perceiving a center of rotation at the fixated object (O5). Although it is difficult to say that the structural rigidity will be maintained during these rotations from the disparity-eccentricity relationship alone, we know that perceptually rigidity is maintained under this condition, perhaps with help from other visual cues like motion parallax or perspective projection.

If a viewer makes a series of head position changes, vestibular signals, as well as other sensorimotor systems, signal the shift of the self-position in the same direction. At the same time, the head shifts cause a series of view changes in the opposite direction. However, since the viewer is aware that self-motion is causing the perspective changes, he/she counterbalances the visual rotation signal and maintains a perception that the world is stable. A shift of perception from object-motion to self-motion, based on additional sensory evidence of self-stability, was also demonstrated with vibration-induced eye movements (Peli & Garcia-Perez, 2003).

The foregoing analysis also supports the binocular depth perception hypothesis suggested by van Ee and Erkelens (1996), which provided results similar to those shown here, and concluded that the perceptual stability of a 3D scene under head and eye movements relies on classes of disparity changes that are invariant with self-motion.

The integration of information regarding self-motion/object-motion is not limited to low-level sensory cues (e.g., binocular disparity, muscle motion, vestibular stimulation, auditory signals), and the cues must be consistent with high-level rules of perception. For example, the angular size of objects should follow the change of egocentric distance, and a new view, which was not visible (occluded) before, should be acquired as viewing perspective changes. If visual information on self-motion/object-motion is not internally consistent, the conflicting signals may provoke motion sickness. Figures 4 and 5 indicate that stereo disparity-based observations in the real world are free from such conflicts.

We now compare the disparity/eccentricity structure of natural stereo viewing (Figures 4 and 5) to ones representing S3D viewing, and show how the resulting differences represent unnatural dynamics in the S3D viewer's perspective (assuming that the disparity/eccentricity relationships in natural viewing are used to provide the perception that a rigid world is rigid).

## 4 Stereoscopic image capture

**Figure 6.**
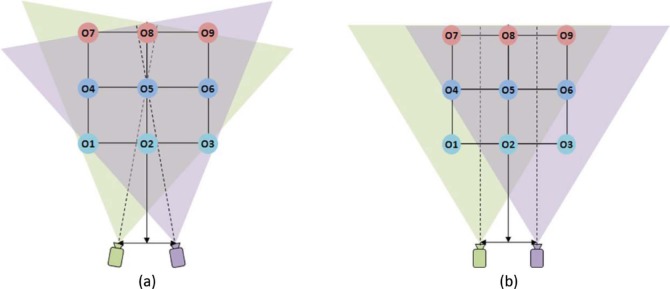
Schematics of stereoscopic scene capture methods. (a) Capture with vergence. (b) Capture without vergence (parallel). The distance between the two cameras is assumed to be 0.06 m, objects O1–O9 are placed on a 1 m grid, and the distance to the first row of the grid from the camera/viewpoint plane is 2 m, as in Figure 2. Diagrams are not to scale. The sensors in the camera are assumed to be placed perpendicular to the camera-aiming direction.

To generate two different views, two cameras (in real-world video) or two viewpoints (in computer graphics) are typically used to capture/render corresponding left and right eyes scenes. Whether it is real-world video recording or computer rendering, there are two common scene capturing configurations (Mendiburu, 2012): one with vergence (converged-axes or “toe-in” stereoscopic imaging) and the other without vergence (parallel-axis stereoscopic imaging) (Figure 6).

When the camera/viewpoint simulates a viewer fixating at center object O5, if the stereo scene is captured with vergence (Figure 6a), the center object is already aligned at the center of the captured images on both cameras. In this case, no additional scene alignment is needed, but it is necessary to rotate the image planes when the captured scene is projected to the screens in order to replicate the angular relationships in the physical scene between the objects and the cameras' planes. Such tilt is not practical in projection or single-monitor stereo display systems. A “lens tilt” design, wherein lens and sensor were independently rotated inside the camera hardware (Kaneko, Suyama, & Yamamoto, 2010), may be used to match the geometry of the capture plane to the display geometry, but the utility of the lens-tilting method is limited, because the tilted lens causes blur. In dual display stereo systems such as HMDs and Wheatstone (mirror-based) stereoscope, it is possible to tilt the display systems and match the capture system's geometry.

If the stereo scene is captured without vergence simulation (Figure 6b), the fixated center object, O5, will be captured right of center in the left camera, and left of center in the right camera. Therefore, the fixated object in the captured images needs to be shifted toward the center of the screen (left image shifted left; right image shifted right) to eliminate the incorrect (nonzero) VE of the target object. The image shift problem can also be addressed during image acquisition by employing a “shift sensor” design, wherein the sensor is horizontally shifted (relative to the camera lens) during capture to center the target object on the sensor (Fehn, 2004).

### 4.1 Capturing the scene with vergence (“Toe-in”)

Since this capture method mimics the extraocular motor control of the human binocular vision system (compare Figures 1 and 6a), the VEs and ADs of the objects in the visual field are naturally preserved in the captured 2D image space. If the captured images are properly projected to viewer's retinas, they should provide the viewer the same disparity configuration (AD–VE relationships) as the real scene, and maintain stable disparity structure throughout camera movements, such as panning and tracking, as observed with natural human binocular vision illustrated in Figures 4 and 5, respectively. However, as mentioned above, the projection angles of the images have to be matched with the vergence angle of the cameras, which is hard to achieve in most commercial display systems and, to my knowledge, has not been implemented where it is possible. This causes unnatural depth distortion with horizontal motion, as will be discussed in greater detail below (Section 5.1).

### 4.2 Capturing the scene without vergence

When the scene is captured without vergence, both cameras are aimed parallel to the cyclopean line of sight that connects the midpoint between the cameras and the target object (Figure 6b). The VEs of other objects while aiming at one object can be computed by subtracting the angle of the cyclopean view (origin to object) from the angle of the camera view (center of camera to object). The AD of the object is, again, the angular difference between VEs captured by the left and right cameras.

**Figure 7.**
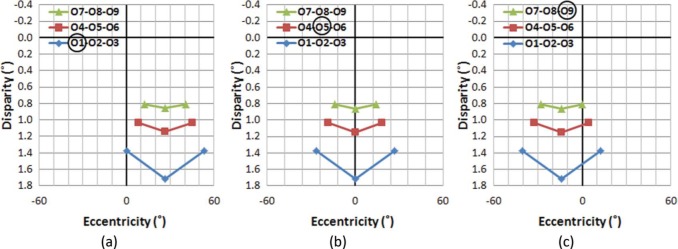
AD distribution of objects in the sample scene when aiming at different objects in the scene, captured by a stereoscopic camera without vergence (“parallel axis”). (a) Aiming at O1, (b) at O5, and (c) at O9. While VEs change with aim, the AD distribution remains constant regardless of camera rotations. Note that the target objects have nonzero ADs.

Figure 7 shows the captured AD distributions, when the camera aims at objects in different rows and columns. Although the VEs of objects vary depending on which object is aimed at, the ADs of all objects do not change with camera rotations (ignoring the slight distance shifts of yoked rather than individually rotated cameras). For example, the VE of the object O5 changes from 26° to 0°, then to −14° as the aim of camera changes from O1 to O5 to O9, but the AD of O5 is kept constant (1.18°) throughout the aiming changes. Therefore, this capturing method produces absolute AD information of objects in the scene (or the relative AD information with respect to an object at infinite distance). This may explain why absolute disparity is frequently considered in display literature rather than the physiologically more relevant relative disparity.

For the human visual system (as well as for camera capture with vergence), other objects' ADs are normalized with respect to the fixated object's zero AD. This effectively limits the range of ADs required for binocular fusion around the attended distance. However, when capturing without vergence, the AD of objects at an infinite distance becomes zero, and the AD increases for objects located closer to the camera. Therefore, an object close to the camera may have a relatively large AD; so large that it may be too large for binocular fusion. For example, the objects on the nearest row (O1-O2-O3) which are around 2 m away from the cameras have ADs larger than 1.4°.

The AD threshold for human binocular fusion depends on several factors, including exposure duration, size, and eccentricity of the stimulus (see Howard & Rogers, 1995 for review), but it is generally accepted that ADs less than 0.5° can be fused easily and quickly, while ADs as large as 1° can be fused with the help of motor vergence and longer exposure time. Therefore, for the purpose of stereo displays, the common binocular fusion AD is kept to less than 1° (Iwasaki, Kubota, & Tawara, 2009). If an object's AD is larger than 1°, fusion can fail and it may be seen as diplopic. In our sample 3D world, when captured without vergence, objects on the first (O1-O2-O3) and likely the second (O4-O5-O6) row will be perceived as doubled.

To avoid the problem of fusing large ADs, horizontal image translation (HIT) is commonly applied (Broberg, 2011; Mendiburu, 2012; Stevenson & Cobian, 2009), so that the aimed object captured by each camera is shifted to the center of the dichoptic images after the capture process. The amount of shift required during HIT depends on the distance to the aimed object, with larger shifts needed for closer objects. The original images need to be wide enough to preserve the desired display size after being cropped to exclude portions available from just one camera. Therefore, applying HIT with motion picture videos has to be followed by cropping and resampling of the images, so that the cropped image size remains constant (Mendiburu, 2012). Note that although HIT is designed to preserve natural AD structure, the amount of shift is often retained as an artistic decision to be made during the postproduction process, which affects the overall (absolute) depth effects of the scene.

**Figure 8.**
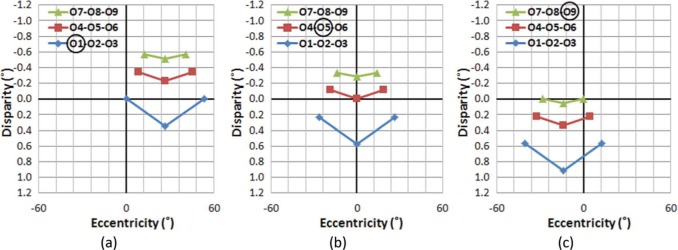
AD distribution of objects in the sample scene when aiming at different objects in the scene, captured by a stereoscopic camera without vergence, but after horizontal image translation (HIT). (a) Aiming at O1, (b) at O5, and (c) at O9. Although the absolute ADs change as a result of aiming changes, the relative AD distribution remains constant. Note the difference in range of the disparity (vertical) axes compared with Figure 7.

Once the aimed object images are aligned, the VEs and egocentric distance of other objects can be computed with the functions given in Equations (1)–(3). Figure 8 shows the resulting AD distribution after HIT, assuming that the projection screen is 3 m away (the physical distance to the center of the object grid). Since HIT is a linear (not angular) transformation, this process inevitably also alters relative AD information. However, the amount of AD distortion is too small to be perceptible (less than 0.00001° AD distortion for the large VE objects in our sample scene).

Comparing the pre-HIT disparity distributions (Figure 7a–c) with post-HIT disparity distributions (Figure 8a–c) reveals that the HIT effectively reduces the maximum disparity to a manageable level (less than 1°), and restores the proper behavior of the (relative) disparity structure, where the aimed object's disparity is zero throughout aiming changes, just as was shown for human binocular fixation changes (Figures 4 and 5). While slight VE differences can be noted when comparing the results of Figure 8 with those of Figure 4, the AD differences are indeed negligible.

## 5 Perception of motion in depth in projected stereoscopic imagery

Regardless of which capture method is used, captured stereoscopic images (pairs of left and right camera/viewpoint perspectives of a scene) are presented separately to viewer's left and right eyes using various methods, such as anaglyphs with red-cyan glasses, active shutter glasses with temporally alternating images, passive polarized glasses with polarized images, or autostereoscopy using lenticular arrays without any glasses. In most common stereoscopic video viewing systems, two images (left and right eye views) are displayed on a single screen, and a viewer watches from a fixed location. In this section, we show how displaying the captured S3D images on a single surface affects binocular disparities and eventually causes perceptual depth distortion. When the two images are displayed on two different displays as in head mounted stereo displays, or in a mirror (Wheatstone) stereoscope, the two display planes are always parallel (though they could be tilted), resulting in similar effects and distortions.

### 5.1 Impact of camera or object movement on motion perception

If the stereoscopic images are captured with vergence simulation (or captured without vergence simulation and then HIT post-processed), they maintain the disparity structure of the scene during camera aiming changes. In natural viewing, absolute disparity varies based on the physical distance to the aimed object (Figure 4). If the viewing position relative to the display or screen is synchronized with the actual distance to the aimed object, the whole 3D world should be perceived as if the viewer is looking at the scene from the camera position. However, it is impossible (or at least currently impractical) to produce that sort of variable distance display or screen. The stereoscopic screen or display is located at a fixed distance. If the distance between the viewer and the screen is shorter than the distance between the camera and the aimed object when the scene was recorded, overall disparity increases (both negative and positive), and the overall scene looks deeper than it should be. If the viewer's distance to the display/screen is larger than the capture distance, overall disparity will be reduced and the scene will be appear shallower in depth (Mendiburu, 2012). However, this overall depth distortion is much easier to adapt to because relative angular disparities change globally, compared to the dynamic, spatially variable, depth distortion described below.

In the following illustrations, we will show how conventional projection of the S3D contents continuously introduces perceptual depth motion distortion (contraction or expansion of depth space that varies with eccentricity), which may cause perceptual motion conflicts.

The set of S3D images captured (as in Figure 8) are assumed to be displayed dichoptically on a single plane that is located 3 m away from the viewer (the distance to the middle row of the scene). The viewer-perceived ADs have been calculated based on onscreen position of the objects using Equation (1)–(3), assuming that the IPD of the viewer is the same as the camera separation (0.06 m).

**Figure 9.**
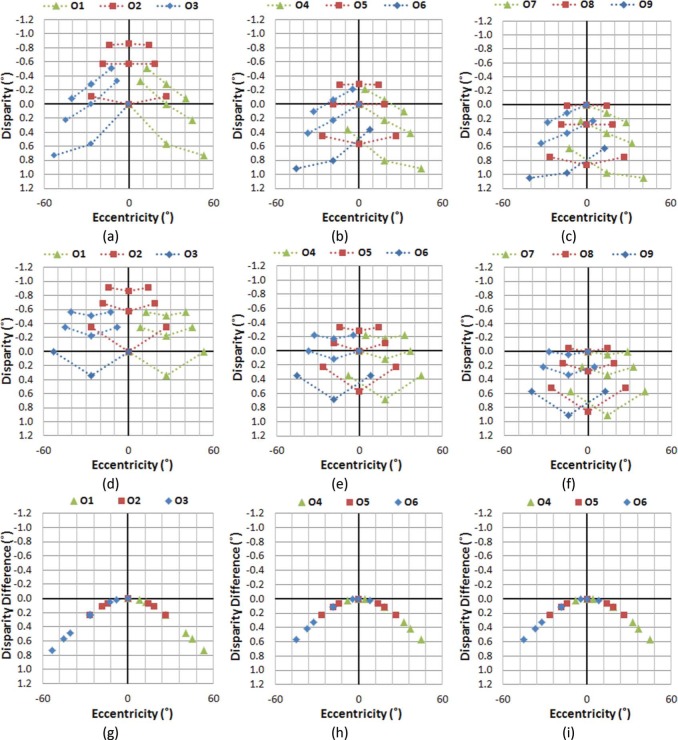
The effect of gaze shifts on AD as a function of eccentricity while the viewer's head remains centered. The ADs of all nine objects are shown for each gaze (fixation) position. In the first two rows (panels a–f), legend symbols distinguish gaze position, not objects rows, with all nine objects per gaze having the same symbol. Panels (a), (b), and (c) show the ADs with S3D viewing, each with three gaze positions overlaid (for fixations on the objects in first row, second row, and third row, respectively). Panels (d–f) show the corresponding ADs during natural viewing. Panels (g–i) plot the arithmetic difference between the S3D and natural ADs as a function of VE, with symbols representing gazed objects, O1–O9. The amount of the depth distortion is largely independent of aiming distance (vergence angle), but is substantial at larger VEs.

Figure 9 illustrates the effect of gaze shifts, while the viewer's head remains centered. Note that if no depth distortion is introduced in the scene capture and projection, the S3D ADs of Figure 9a–c should have the same shape as those for the natural views of Figure 9d–f. However, the S3D scene as perceived by the viewer shows substantial AD distortions (differences from natural viewing, as shown in Figure 9g–i), which unavoidably introduce perceptual changes in apparent depth structure. Even though it is possible to achieve binocular fusion at most of the AD values, the magnitude of the depth distortion at higher eccentricities is substantial.

Comparing Figure 9a–c to 9d–f reveals that the objects displayed at larger VEs get progressively larger ADs than they have in natural viewing. Therefore, the viewer will perceive objects shown at larger VEs to appear closer than they should be. Note that since the S3D images are displayed at a fixed distance (the distance between the camera and the middle row), Figure 9a–c can also represents situations in which a viewer's viewing distance is farther (Figure 9a), the same (Figure 9b), and nearer (Figure 9c) than the captured aiming distance. Still, it is apparent that the shape of the depth distortion is only slightly affected by aiming distance. This indicates that the source of the depth distortion is not directly related to the vergence change during the S3D capture process, but rather due to the rotation of the left and right image planes with respect to the projection screen.

For static S3D images, the impacts of these depth distortions are minimal, for several reasons. First, the brain perceives the structure in conjunction with undistorted 2D monocular depth cues such as linear perspective that serve to counteract the AD distortion. Second, large AD distortions occur at larger VEs (peripheral vision), where the depth perception accuracy is much lower (Prince & Rogers, 1998). Third, the outer VE area gets less attention (even with 2D distortions) and static spatial distortion in the periphery can be easily unnoticed (Bex, 2010).

If there is motion in the scene (or motion of the camera), these disparity distortions continuously introduce changing depth motion distortions, and produce unnatural optic flow, which are nonexistent in the real world. In our sample scene, when a camera scans the scene horizontally (by panning and/or tracking during capture) from object O1 to object O2, and then to object O3 (Figure 9a), the S3D viewer perceives unnatural and varying compression and expansion of the depth space. At the same time, perspective and object size cues indicate no depth change. For example, the viewer's perception of the depth structure will change as if the distance between objects in the left column (O1-O4-O7) is compressed then expanded, while the distance between objects in right column (O3-O6-O9) is expanded then compressed. This would also affect the perceived thickness of objects or structures, conflicting with their known rigidity.

If the camera approaches or retreats to/from the objects at constant speed (Figure 9a to 9b to 9c), the objects at larger VEs will be perceived as if they are speeding up or slowing down, respectively, compared to the objects at central VE that maintain constant speed. In other words, the viewer perceives progressively faster depth motion at larger VEs, which creates an illusion of elongation or compression of rigid bodies in depth at larger VEs. These perceived changes in spatial depth create high-level conflicts for what is expectedly a stable environment, e.g., the ground or rigid walls appearing to expand or contract during camera movements. Note that similar distortions will be produced when objects in the scene change their eccentricity due to their motions, even when the camera aiming is static.

### 5.2 Impact of the viewer's head movements on motion perception

In the previous analysis, the viewer's head position was assumed to be fixed and aligned with center of the projection screen. In reality, viewers' head position frequently changes due to posture adjustments or purposeful movements. In the following illustrations, we will show how the viewer's head motion affects depth perception of the stereoscopic display, and how this causes unnatural motion parallax, which may cause perception of ego- and exo-centric motion conflicts.

In real-world viewing, a viewer's horizontal self-motion while looking at a fixed spot causes motion parallax that is perceived as relative rotation of the depth structure in the opposite direction. For example, if a viewer, watching a static real-world 3D scene, moves rightward, the viewer sees a relative clockwise-rotated view of the scene. The rotational speed of objects is governed by linear perspective rules, so the near objects move faster and the farther objects are move more slowly.

**Figure 10.**
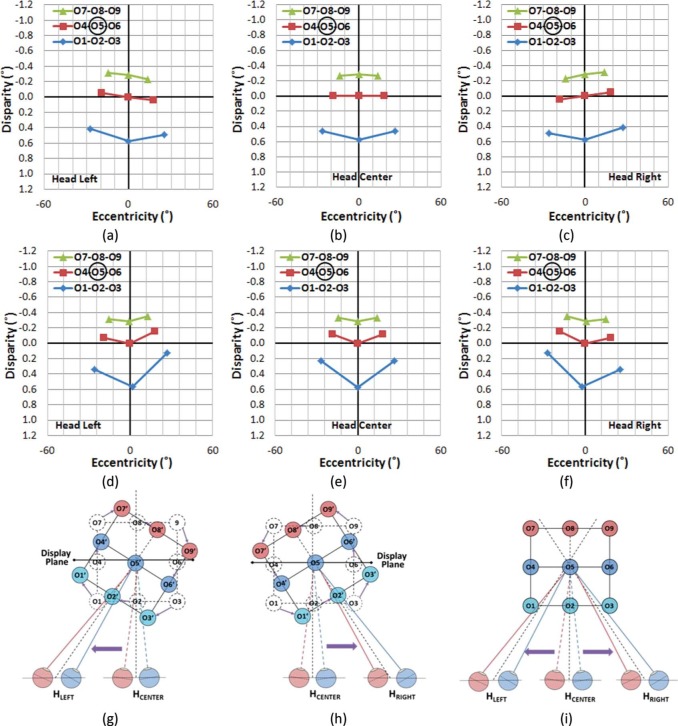
Distribution of angular disparities and visual eccentricities of objects when the viewer's position has shifted −0.2 m, 0.0 m, and 0.2 m from the center while fixating on the center object (O5). (a–c) Viewing S3D. (d–f) Natural viewing. With S3D, as a viewer makes continuous head movements from left to right, (a) → (b) → (c), the viewer will perceive the left side of the scene to be moving closer and the right side of the scene to be moving farther, giving the illusion that the whole scene is rotating counter-clockwise in depth. Thus, the viewer will perceive nonexistent rotational motion in the scene, as it seems to turn to follow and face the viewer. However, the same head movements under natural stereo viewing conditions, (d) → (e) → (f), provide changing views of the scene, which is perceived as the unmoving scene moving clockwise. (g) The resulting perceived depth axis rotation as the head moves left with S3D and (h) as the head moves right. (i) The scene remains stable with the same head movements during natural viewing.

Figure 10a–c shows the perceived AD distribution of the displayed stereoscopic images with viewer's head position at 0.2 m left (a), 0.0 m (b), and 0.2 m right (c) from the center. Viewing distance is the same as in the previous examples (3 m). Corresponding AD distribution for the real-world viewing condition is shown in Figure 10d–f.

When viewing the S3D scene, if the viewer makes head shifts from the left (Figure 10a) to the center (Figure 10b), and then to right (Figure 10c), the AD distribution change indicates that the viewer will perceive the objects on the left side (O1-O4-O7) to be moving closer to the viewer, and the objects in the right (O3-O6-O9) moving farther away. Therefore, the viewer will perceive the whole AD structure as turning counter-clockwise. With natural stereo viewing, as it is shown in Figure 10d–f, the perceived direction of objects' rotation in depth would be reversed, and the depth structure appears to rotate clockwise relative to the viewer. Note that the amounts of head shift shown in Figure 10d–f are exactly the same as in Figure 10a–c, but due to the depth motion distortion in the periphery (as described in Section 5.1), the reversed rotation of the scene will be relatively exaggerated.

It can also be observed that the VE values of the center column objects (O2-O5-O8) during S3D viewing (Figure 10a–c) are kept almost constant, even when the viewer's head position is shifted. However, in natural stereo viewing (Figure 10d–f), the VE values of the center column objects are shifted slightly off-center; O2 moves left and O8 moves right when the viewer's head moves left, and O2 moves right and O8 moves left when the viewer's head moves right. At the center head position, O2 would block the view of O5 and O8, while all three would be visible with head shifts in natural stereo viewing. With S3D, they remain aligned with zero AD, so the percept is that they have rotated to maintain that alignment as the head is shifted (O2 would block the view of O5 and O8, regardless of head position). The occlusion cue is possibly the strongest cue and can easily overcome a competing stereo cue.

The combined effect of these perceptual changes in ADs and VE in response to the viewer's head movements induces a perceptual illusion that the whole depth structure is rotating, with the center of rotation at the display (zero disparity) plane. However, during natural stereo viewing, if a viewer's head position changes while looking at an object, the perceived center of rotation is at the position of the gazed object.

Thus, when a viewer's head moves laterally when viewing S3D, several perceptual motion conflicts may rise and that could lead to motion sickness. First, due to the AD distortion between left and right sides of the visual fields, the viewer is presented with unequal compression/expansion of the space, left and right of fixated objects. This can make a seemingly rigid depth structure appear to be deformed. Second, the natural motion parallax rule is violated, as objects positioned closer to the viewer from the fixated object fail to move faster than the farther objects in a direction opposite the head motion. The illusion of depth structure rotation is centered at the display plane (not necessarily at the fixated object) and all visible objects move at the same angular speed regardless of their distance, and they move in the same direction as the head motion, not opposite. This causes a dramatic illusion that the presumably stable world imaged in S3D is rotating in a direction that follows the viewer's head position. Although the human vision system can compensate for an incorrect viewing angle up to 30° with 2D pictures and still maintain perceptual invariance, compensating for the 3D display distortions associated with changes in viewing position (Banks, Held, & Girshick, 2009) becomes much more complex due to the dynamic 3D distortion explained above.

In addition to this low-level perceptual effect, there is also a high-level perceptual motion conflict. The translational motion of the viewer from one side to another under natural conditions is always accompanied by corresponding view changes that expose new aspects of an object (dynamic disclosure) and occlude others (dynamic occlusion) (Patterson & Silzars, 2009). This view change is not limited to the view of a single object. It extends to the visibility of foreground-background depth relations (visual parallax). However, since the S3D scene as displayed and perceived by the viewer has only one view (the camera viewpoint), a viewer's head movements do not provide the expected visual parallax changes (absence of multi view and dynamic occlusion and disclosure). The lack of changing perspective in spite of the physiological sensation of head motion makes it easier for viewer's brain to interpret the view-following illusion as a result of the objects moving to track the viewer's head movements. This is similar to the rotational illusions seen with the hollow-face illusion (Hill & Bruce, 1993) and Patrick Hughes's reverspective drawings (Papathomas & Bono, 2004).

## 6 Discussions

We have shown that the viewer's eye movements do not affect the relative angular disparity (AD) during natural viewing of a 3D (real-world) scene (Figures 4a–c and 5a–c). It is evident that although the absolute ADs do change with changes in the viewer's fixation point, the relative AD relationships among objects are maintained throughout the viewer's gaze movements. The perceived depth structure in real-world scenes does appear to be stable, supporting the primacy of relative depth for perception in stereo displays (Peli et al., 2001; van Ee & Erkelens, 1996). This notion of a stable visual world is a key to maintaining body position and making judgments about self and external motions (Prothero & Furness, 1998).

However, regardless of which video capture method (converging or parallel) is used, depth distortions exist in conventional S3D display configurations, and can be computed as AD differences between what would be perceived in the real world and what is perceived from the projected S3D images (shown as the curved lines in Figure 9g–i). If the projected scene is stationary, the impact of depth distortions may be minimal. However, if the scene contains any kind of motion, whether it is caused by camera motion (panning/tracking) or object motion that moves the objects to different visual eccentricities, complex interactions of depth expansion, and compression make the motions in the displayed S3D scene appear unnatural, where the local and global motions (e.g., peripheral vs. central vision) are in conflict.

In real-world conditions, perceived depth changes of objects naturally accompany changes in retinal size, but the relative ADs will be kept consistent, even with eye movements. However, if motion of the camera viewpoint is accompanied by spatial (depth) distortions that are unique for S3D viewing, the perception of relative distance among objects (in addition to the absolute distance to the viewer) in the S3D scene will break in an unexpected way (even for what should appear as a rigid body). Then, the viewer will perceive compression or expansion in depth (*depth wobbling*), while other visual cues, such as perspective and size, show no depth change. These dynamic spatial distortions cause visual-to-visual cue conflicts, likely to be a cause of motion sickness reported with S3D movies.

Furthermore, in real-world viewing, when a viewer's head position is shifted laterally while fixating on an object, the depth structure appears to be rotated in the opposite direction of the viewer's head movement (Figure 10d–f), but the perception of the depth structure remains stable because of the compensational vestibular signal that registers the ego-motion and counters the exo-motion. However, when the same scene is viewed in an S3D display (Figure 10a–c and g–i), the viewer's lateral position shift introduces a rotation of the depth structure centered at the display plane axis that follows the viewer's viewing angle toward the screen/display plane. As a result, it creates an illusion that the whole 3D depth structure appears to be rotating in the same direction as viewer's head movements (Figure 10g–i). This rotation of the viewing angle may cause strong external artificial motion because the same amount of angular rotation is applied to the whole 3D depth structure, regardless of how far or close the real objects are located. This type of shift is inconsistent with the motion parallax experienced naturally.

The problem becomes more severe if other visual cues indicate that the scene is stationary. The motion parallax rules predict that closer objects make a large retinal slip and show more of the preoccluded view of the object, and farther objects create a small retinal slip, so that they are perceived as a relatively stationary background per linear perspective rules. Since the S3D image only shows a single view from a particular viewpoint (the camera aiming direction) that is independent of the viewer's egocentric motions, the lateral changes of the viewer's viewpoint do not provide any additional view of the near objects. This creates an illusion of unnatural rotation, where the viewer's brain expects to see larger view changes of the near objects and the less retinal slip for the farther objects, but the whole scene appears to make no view changes and large retinal slips.

All of the visual conflicts mentioned above can be sources of the S3D-induced motion sickness. However, viewers may not easily adapt to those conflicts, first because the amount and direction of the perceived 3D motion distortions change continuously as scene content changes. Second, it is hard to adapt to the randomness of the head movements that initiate motion parallax conflicts. Third, the type of perceptual motion conflicts related to depth rarely exist when viewing naturally.

Studies of 3D visual field stimulation (Piponnier, Hanssens, & Faubert, 2009), which measures body sway amplitude when a subject is exposed to sinusoidal motion of a tunnel, showed that postural stability is significantly more affected by the peripheral visual field (VE > 7°) compared to central vision. Since most of the motion distortions due to spatial (depth) distortions in S3D image perception occur in the periphery, it can be expected that the impact of the depth motion distortion in the periphery is more severe than central distortions. Faubert and Allard (2004) also measured body sway amplitude when subjects were exposed to a simulated dynamic visual distortion similar to that caused by progressive ophthalmic lenses, and showed that the postural instability increases more when the stimulus moves laterally rather than vertically. Although the study relied on distorted 2D projections of a scene (nonlinear 2D stretching toward bottom corners) and did not directly measure the level of motion sickness, increased instability of the posture is an indication that vision-to-vision motion signal conflicts can disturb postural stability and may cause motion sickness.

When an S3D scene is captured with vergence, the target object in the scene is already aligned in the center of each screen, so that no other alignment is necessary (e.g., Panasonic HDC-Z10000). However, since the captured screens are displayed on a single screen, the projection process introduces a rotation of each image in-depth at half of the convergence angle. This observation provides a clue for how the distortion can be eliminated. If two separate stereoscopic displays are used, as in head-mounted display systems, or a Wheatstone stereoscope, the left and right view images need to project onto two rotated projection planes that match the camera vergence angles to maintain correct perspective of captured scene. However, although active camera systems that vary convergence with focus controls exist (e.g., the GS-TD1B from JVC, Yokohama, Japan), an active projection/display that rotates in real-time is currently not practical. Therefore, it may be preferable and more practical to use image processing techniques (spatial remapping) to obtain the same correctional varying effects.

The correction for projection planes should be similar to the method that is commonly used to correct the well-known keystone distortion in stereo images. The keystone distortion is a 2D geometric distortion, where the left edge of the image captured by the left camera is taller than right edge, and the right edge of the image captured by the right camera is taller than left edge when they are perceived. Keystone distortion is known to cause difficulty fusing stereoscopic imagery and may induce additional eye strain (Peli, 1999; Woods et al., 1993). To correct keystone distortion, single linear scaling of height along the lateral direction is applied, so that the heights in left and right images are identical. Note that this does not correct the AD (depth) distortion. To correct the depth distortion caused by the fixed projection planes, nonlinear scaling of width along the lateral direction should be applied to each image, so that the corresponding virtual projection planes are rotated based on the vergence angle of the image and the distance between the viewer and the projection/display (real or virtual) screen. These correction will address distortion due to camera or object motion but not due to viewer's head motion.

The perceptual motion conflicts caused by viewer-induced motion (parallax distortion) can be corrected by tracking the viewer's head position. This approach can be a direct solution when there is only one viewer watching the scene, and the scene is computer-generated graphics, so that new views can be computed. However, there are limitations to this approach when the stereoscopic video is shared by multiple viewers. Tracking multiple heads and synthesizing the scene with proper perspective may be hard to achieve in real time, and although separate perspective scenes can be generated for multiple viewers, projection/display technology to deliver multiple viewer-specific views in a precise manner is hard to achieve. Recent developments to provide different views at different viewing angles (multi-view display) are still in early stages and have many limitations due to the restricted viewing angles, crosstalk, and lack of high bandwidth for video production and data conveyance infrastructure (Jung et al., 2011; Park et al., 2009).

## 7 Conclusions

As shown throughout this paper, it is critical to expand the analysis of 2D geometrical distortions introduced during the capture and projection to the analysis of the S3D video perceived by the viewer, so that the depth illusion from the viewer's perspective can be analyzed and understood. Perceptual invariance achieved when watching 2D pictures is not sufficient for models of viewing S3D video, as S3D to 3D perceptual effects are still in need of more empirical data and theoretical analysis to support prediction of the perceptual and concomitant physiological effects.

Our proposed source of motion sickness symptoms in S3D displays is purely analytic, and the analysis is based on simple epipolar geometry (Hartley & Zisserman, 2003). Empirical confirmation is required to determine if those postulated sources that we have identified are really responsible for motion sickness, and if so, it would be important to measure the relative weights and interactions among those sources, creating a quantitative model of 3D motion sickness invocation based on motion in video content and viewer's self-motion, and creating a compensational algorithm to reduce the sickness inducing factors.

A variety of questionnaires, such as the Simulator Sickness Questionnaire (SSQ) (Kennedy, Lane, Berbaum, & Lilienthal, 1993), have been used to measure the subjective level of motion sickness that the subjects report after certain exposure durations to the S3D stimuli, and objective physiological measurements such as body sway amplitude, blood pressure, body temperature, sweating, change of skin conductivity, heart rate variability (Holmes & Griffin, 2001), electrogastrography (EGG) (Cheung & Vaitkus, 1998; Hu, Grant, Stern, & Koch, 1991), and electroencephalography (EEG) (Chen et al., 2009; Wood, Struve, Straumanis, Stewart, & Wood, 1991) have been proposed and successfully demonstrated as surrogate measures for the dynamics of motion sickness. Some of these may be useful in further investigation of our proposed S3D motion sickness causes, which will be demonstrated best by comparing the same S3D system and contents with and without the corrective measures we are proposing.

The possible sources of visually induced 3D motion sickness discussed here, the spatial (depth) distortions and rotation of the depth axis, should open various approaches for reducing the discomfort associated with viewing these videos. Since motion sickness is one of the most critical drawbacks of the S3D visual experience, identifying the causes of this problem will be the foundation for the further research into its mitigation.
